# Weight gain among treatment‐naïve persons with HIV starting integrase inhibitors compared to non‐nucleoside reverse transcriptase inhibitors or protease inhibitors in a large observational cohort in the United States and Canada

**DOI:** 10.1002/jia2.25484

**Published:** 2020-04-15

**Authors:** Kassem Bourgi, Cathy A Jenkins, Peter F Rebeiro, Frank Palella, Richard D Moore, Keri N Altoff, John Gill, Charles S Rabkin, Stephen J Gange, Michael A Horberg, Joseph Margolick, Jun Li, Cherise Wong, Amanda Willig, Viviane D Lima, Heidi Crane, Jennifer Thorne, Michael Silverberg, Gregory Kirk, William C Mathews, Timothy R Sterling, Jordan Lake, John R Koethe, for the North American AIDS Cohort Collaboration on Research and Design (NA‐ACCORD)

**Affiliations:** ^1^ Vanderbilt University Medical Center Nashville TN USA; ^2^ Indiana University School of Medicine Indianapolis IN USA; ^3^ Northwestern University Feinberg School of Medicine Chicago IL USA; ^4^ Johns Hopkins University Baltimore MD USA; ^5^ University of Calgary Calgary AB Canada; ^6^ National Cancer Institute Bethesda MD USA; ^7^ Mid‐Atlantic Permanente Research Institute Kaiser Permanente Mid‐Atlantic States Rockville MD USA; ^8^ Centers for Disease Control and Prevention Atlanta GA USA; ^9^ University of Alabama at Birmingham Birmingham AL USA; ^10^ University of British Columbia Vancouver BC Canada; ^11^ University of Washington Seattle WA USA; ^12^ Kaiser Permanente Division of Research Kaiser Permanente Northern California Oakland CA USA; ^13^ University of California San Diego San Diego CA USA; ^14^ University of Texas Health Science Center at Houston Houston TX USA; ^15^ Veterans Affairs Tennessee Valley Healthcare System Nashville TN USA

**Keywords:** integrase inhibitors, weight gain, obesity, metabolic, HIV, North America

## Abstract

**Introduction:**

Weight gain following antiretroviral therapy (ART) initiation is common, potentially predisposing some persons with HIV (PWH) to cardio‐metabolic disease. We assessed relationships between ART drug class and weight change among treatment‐naïve PWH initiating ART in the North American AIDS Cohort Collaboration on Research and Design (NA‐ACCORD).

**Methods:**

Adult, treatment‐naïve PWH in NA‐ACCORD initiating integrase strand transfer inhibitor (INSTI), protease inhibitor (PI) or non‐nucleoside reverse‐transcriptase inhibitor (NNRTI)‐based ART on/after 1 January 2007 were followed through 31 December 2016. Multivariate linear mixed effects models estimated weight up to five years after ART initiation, adjusting for age, sex, race, cohort site, HIV acquisition mode, treatment year, and baseline weight, plasma HIV‐1 RNA level and CD4^+^ cell count. Due to shorter follow‐up for PWH receiving newer INSTI drugs, weights for specific INSTIs were estimated at two years. Secondary analyses using logistic regression and all covariates from primary analyses assessed factors associated with >10% weight gain at two and five years.

**Results:**

Among 22,972 participants, 87% were male, and 41% were white. 49% started NNRTI‐, 31% started PI‐ and 20% started INSTI‐based regimens (1624 raltegravir (RAL), 2085 elvitegravir (EVG) and 929 dolutegravir (DTG)). PWH starting INSTI‐based regimens had mean estimated five‐year weight change of +5.9kg, compared to +3.7kg for NNRTI and +5.5kg for PI. Among PWH starting INSTI drugs, mean estimated two‐year weight change was +7.2kg for DTG, +5.8kg for RAL and +4.1kg for EVG. Women, persons with lower baseline CD4^+^ cell counts, and those initiating INSTI‐based regimens had higher odds of >10% body weight increase at two years (adjusted odds ratio = 1.37, 95% confidence interval: 1.20 to 1.56 vs. NNRTI).

**Conclusions:**

PWH initiating INSTI‐based regimens gained, on average, more weight compared to NNRTI‐based regimens. This phenomenon may reflect heterogeneous effects of ART agents on body weight regulation that require further exploration.

## 1 INTRODUCTION

Weight gain following antiretroviral therapy (ART) initiation is common among persons with HIV (PWH), particularly those with more pronounced CD4^+^ cell count (CD4) depletion or lower pre‐ART body mass index (BMI) [1, 2, 3]. In the early ART era, weight gain on treatment was often seen as evidence of nutritional rehabilitation and was associated with improved survival and immunologic recovery [3, 4, 5]. However, over the past two decades the BMI of PWH on ART has steadily increased. In one multisite United States (US) study, over half of those remaining on treatment at three years were overweight or obese [1]. Among PWH, a high BMI is associated with an increased risk of developing diabetes, neurocognitive impairment, and other comorbid conditions [6, 7, 8, 9], and the avoidance of weight gain may reduce these risks.

Integrase strand transfer inhibitors (INSTI; e.g. raltegravir (RAL), elvitegravir (EVG), dolutegravir (DTG) and bictegravir (BIC)) are a more recently available class of antiretroviral medications with a good tolerability profile and, for DTG and BIC, a substantial genetic barrier to the emergence of HIV drug resistance [10, 11]. INSTI‐based ART regimens are now the recommended first‐line treatment for most PWH [12], but several recent studies, primarily from single sites or cohorts, report greater weight gain among persons receiving INSTI‐based ART regimens for initial therapy as compared to protease inhibitor (PI) and non‐nucleoside reverse transcriptase inhibitor (NNRTI)‐based regimens. In a cohort from Brazil, PWH receiving RAL‐based regimens were sevenfold more likely to become obese (BMI ≥ 30kg/m^2^) compared to those receiving NNRTI‐ or PI‐based regimens [13]. In other observational studies, INSTI‐based regimens generally, and particularly DTG‐based ART regimens [14, 15, 16, 17], were also associated with greater weight gain. In the prospective AIDS Clinical Trial Group (ACTG) study A5257, the use of RAL with tenofovir disoproxil fumarate and emtricitabine (TDF/FTC) as an initial ART regimen was associated with greater increases in waist circumference and increased odds of a >10% weight gain at 96 weeks, compared to ART regimens including ritonavir‐boosted darunavir or ritonavir‐boosted atazanavir, each combined with TDF/FTC [18, 19].

ART‐associated weight gain among PWH may contribute to an increased risk of cardiometabolic disease, but the magnitude of weight gain associated with use of different initial ART regimens has varied in recent studies [14, 20]. Given the clinical implications of higher body weight on long‐term health and the broad adoption of INSTI‐based ART regimens as recommended first‐line therapy, further data are needed describing weight trajectories among large numbers of PWH starting different ART regimens and who are diverse in terms of sex, race/ethnicity and age. Herein, we use the North American AIDS Cohort Collaboration on Research and Design (NA‐ACCORD), which pools longitudinal data from multiple regions of the United States and Canada, to conduct a rigorous analysis of weight changes over time and investigate risk factors for weight gain among over 20,000 treatment‐naive PWH who initiated ART.

## 2 METHODS

NA‐ACCORD, one of the regional cohorts supported by the International epidemiologic Databases to Evaluate AIDS (IeDEA) consortium sponsored by the National Institutes of Health, is a multi‐site collaboration involving 26 cohort studies representing over 200 clinical and research facilities in the United States and Canada [21]. PWH are eligible to be enrolled in the NA‐ACCORD from contributing cohorts if they have ≥2 clinical visits within 12 months. NA‐ACCORD collects individual‐level standardized data on demographic and clinical characteristics, ART use, laboratory measurements, medical diagnoses and vital status. These data are pooled, harmonized and undergo quality control procedures for accuracy and completeness at the central Data Management Core (University of Washington, Seattle, WA, USA), followed by additional quality control procedures and assembly into analytic‐ready summary files at the Epidemiology/Biostatistics Core (Johns Hopkins University, Baltimore, MD, USA). Submission of data to NA‐ACCORD requires institutional review board (IRB) approval at each participating site, and as data are de‐identified (except for dates) a waiver of consent is obtained. The review and approval to conduct this specific NA‐ACCORD analysis was provided by the Vanderbilt University IRB.

For this analysis, we utilized NA‐ACCORD data on ART‐naive adults who initiated a first regimen that contained an NNRTI, PI or INSTI in combination with dual nucleoside/nucleotide reverse transcriptase inhibitors (NRTIs) between 1 January 2007 and 31 December 2016, and had available baseline weight and height information. The date of ART initiation was considered the first occurrence of sustained ART use for more than 45 days. Participants were classified as ART‐naive if there was no recorded ART exposure of more than 45 days prior to the start of a combination NNRTI‐, PI‐ or INSTI‐based regimen (this included review of all available records prior to 2007) and the viral load at treatment initiation was detectable (≥400 copies/mL). Women who had previously received ART in the antenatal period were considered “naïve” only if their prior ART exposure was for <45 days.

The primary aim of this analysis was to assess the relationship between ART class and weight over time among treatment‐naïve participants. Analyses were conducted separately among: (1) the full group of participants who met inclusion criteria, where initial ART was categorized by class as NNRTI‐, PI‐ or INSTI‐based; (2) the sub‐group of participants who initiated an INSTI‐based regimen, categorized as RAL, DTG or EVG; and (3) the sub‐group of participants who initiated either an NNRTI‐ or PI‐based regimen, or RAL, DTG or EVG. For each analysis, NA‐ACCORD sites with fewer than 50 PWH who initiated each ART class/INSTI drug were excluded from the analysis. Additionally, participants on regimens containing more than one major ART class (e.g. NNRTI and INSTI) were also excluded from the analysis.

Demographic and clinical characteristics were summarized across the ART/drug categorization using median (interquartile range) or percent with frequency, as appropriate. Kruskal‐Wallis tests assessed the associations of continuous variables with the ART class/drug; Pearson’s chi‐squared tests assessed association between categorical variables and ART class/drug.

We modelled weight changes over time using multivariate linear mixed effects models treating each participant as a random effect. Participants were censored at the time of a switch to a new ART class (i.e. not including NRTI switches or within‐class switches) or at the first instance of virologic failure. Virologic failure was defined as a plasma HIV‐1 RNA >1000 copies/mL, or at the first of two consecutive detectable HIV‐1 RNA measurements ≥400 copies/mL after previously being virologically suppressed [12]. Models adjusted for ART class/drug (not including NRTI backbone), years since ART initiation, sex assigned at birth, self‐reported race, HIV risk factor, age, baseline weight, baseline CD4 (square‐root transformed), baseline HIV‐1 RNA (log_10_‐transformed), cohort site and year of ART initiation. Interactions between ART class/drug and years since ART initiation, sex, race and HIV risk factor were assessed. To relax linearity assumptions, years since ART initiation, age, and baseline weight, CD4 and HIV‐1 RNA were modelled using restricted cubic splines with five knots. Baseline weight and CD4 were the values closest to ART initiation within a window of −180 to +30 days. Baseline HIV‐1 RNA was the value closest to ART initiation within a window of −180 to +7 days. Self‐reported race was dichotomized as white versus non‐white. HIV risk factor was classified as men who have sex with men (MSM), persons who inject drugs (PWID, including MSM/PWID), heterosexual and other/unknown. Missing baseline data were multiply imputed using 10 imputation replications. Marginal predicted weights over time along with 95% bootstrapped confidence intervals (CIs) were calculated by ART class/drug and the predicted weights at two and five years after ART initiation were reported.

In secondary analyses, to further explore the relationship between weight over time and ART use, we assessed weight gain in excess of 10% at two and five years post‐ART initiation by regimen class, and at two years by individual INSTI medications. Weight at two and five years was defined as the weight closest to those time points and within a window of ±6 months. Persons with missing baseline and follow‐up weight were excluded from these secondary analyses. Using >10% weight gain as an outcome, we used multivariate logistic regression adjusting for the same covariates used in the primary analysis. As in the primary analysis, continuous variables were fit with restricted cubic splines using five knots; however, in this analysis no interaction terms were included. Missing data were multiply imputed with 10 imputation replications.

Lastly, we evaluated the proportion of participants who transitioned between the standard BMI categories of normal (18.5 to 24.9kg/m^2^), overweight (25.0 to 29.9kg/m^2^) and obese (≥30kg/m^2^) after three years of ART. Analyses were performed using R (version 3.4.4; http://www.r-project.org).

## 3 RESULTS

A total of 22,972 ART‐naive PWH who started treatment between 2007 and 2016 and met inclusion criteria were available from 13 cohorts in NA‐ACCORD. The study population was 87% male and 41% white. Median age at ART initiation was 42 years, BMI 25kg/m^2^, CD4 count 308 cells/mm^3^ and log_10_ HIV‐1 RNA 4.6 copies/mL. Table 1A shows baseline characteristics of participants by ART class (NNRTI, PI and INSTI) at treatment initiation. Of the 22,972 PWH, 11,296 (49%) starting NNRTI‐based regimens, 7038 (31%) PI‐based regimens and 4638 (20%) INSTI‐based regimens. PWH initiating INSTI‐based regimens were younger, more likely to report MSM as their probable HIV risk factor, had higher CD4, and initiated ART later (2014) than those initiating NNRTI‐ and PI‐based regimens (2010). The majority of persons across all three groups had tenofovir‐based NRTI backbones, which was predominantly TDF with only 130 participants (0.7%) receiving tenofovir alafenamide fumarate (TAF). The low use of TAF is likely a reflection of the cohort close date of 31 December 2016.

**Table 1:** Baseline clinical and demographic characteristics of (A) all participants by regimen class (INSTI‐, NNRTI‐ and PI‐based regimens) and (B) all participants starting INSTI‐based regimens by medication

Variables		(A)				(B)			
		ART class				INSTI drug^a^			
		NNRTI‐based regimens	PI‐based regimens	INSTI‐based regimens	*p*‐value 1	Raltegravir	Dolutegravir	Elvitegravir	*p*‐value 2
N (%)		11,296 (49%)	7038 (31%)	4638 (20%)		1192 (28%)	926 (22%)	2068 (50%)	
Age at time of ART initiation^b^		43 (32, 52)	42 (32, 50)	39 (29, 50)	<0.001	45 (35, 53)	38 (29, 51)	35 (27, 47)	<0.001
Birth sex	Male	10,214 (90%)	5668 (81%)	4003 (86%)	<0.001	1016 (85%)	795 (86%)	1842 (89%)	0.002
	Female	1082 (10%)	1370 (19%)	635 (14%)		176 (15%)	131 (14%)	226 (11%)	
Race group	White	4658 (41%)	2788 (40%)	1973 (43%)	<0.001	549 (46%)	395 (43%)	865 (42%)	0.18
	Black	4769 (42%)	3054 (43%)	1872 (40%)		475 (40%)	375 (40%)	865 (42%)	
	Hispanic	830 (7%)	587 (8%)	407 (9%)		87 (7%)	80 (9%)	157 (8%)	
	Other	1039 (10%)	609 (9%)	386 (8%)		81 (7%)	76 (8%)	181 (10%)	
Risk group	MSM	4332 (38%)	2127 (30%)	2232 (48%)	<0.001	482 (40%)	520 (56%)	1189 (58%)	<0.001
	PWID^c^	1348 (12%)	1026 (15%)	380 (8%)		152 (13%)	53 (6%)	141 (7%)	
	Heterosexual	1638 (15%)	1411 (20%)	809 (17%)		205 (17%)	207 (22%)	381 (18%)	
	Other	3978 (35%)	2474 (35%)	1217 (27%)		353 (30%)	146 (16%)	357 (17%)	
NRTI backbone	TDF‐based	10,617 (94%)	5476 (78%)	3676 (79%)	<0.001	1056 (89%)	248 (27%)	1932 (93%)	<0.001
	TAF‐based	8 (0%)	0 (0%)	122 (3%)		1 (0%)	7 (1%)	114 (6%)	
	ABC‐based	370 (3%)	830 (12%)	760 (16%)		98 (8%)	653 (70%)	0 (0%)	
	Other	301 (3%)	732 (10%)	80 (2%)		37 (3%)	18 (2%)	22 (1%)	
Baseline CD4 T‐cell count (cells/µL)^b^		313 (180, 452)	262 (105, 406)	361 (198, 533)	<0.001	336 (173, 499)	388 (212, 594)	382 (216, 558)	<0.001
Baseline log HIV‐ RNA (copy/mL)^b^		4.6 (4.0, 5.1)	4.7 (4.1, 5.2)	4.6 (4.1, 5.1)	<0.001	4.6 (4.0, 5.1)	4.6 (4.1, 5.1)	4.6 (4.1, 5.2)	0.075
Baseline BMI (kg/m^2^)^b^		25 (23, 29)	25 (22, 28)	25 (22, 29)	<0.001	26 (23, 29)	25 (22, 29)	25 (22, 29)	0.048
Year of ART Initiation^b^		2010 (2008, 2012)	2010 (2008, 2012)	2014 (2012, 2015)	<0.001	2011 (2010, 2013)	2015 (2015, 2016)	2014 (2014, 2015)	<0.001

*p*‐value 1: comparing the difference in baseline characteristics by regimen class (INSTI‐, NNRTI‐ and PI‐based regimens). *p*‐value 2: comparing the difference in baseline characteristics by specific integrase inhibitors (raltegravir, elvitegravir and dolutegravir).

ABC, abacavir; ART, antiretroviral therapy; BMI, body mass index; INSTI, integrase strand transfer inhibitors; MSM, men who have sex with men; NNRTI, non‐nucleoside reverse transcriptase inhibitors; NRTI, nucleoside/nucleotide reverse transcriptase inhibitors; PI, protease inhibitors; PWID, persons who inject drugs; TAF, tenofovir alafenamide fumarate; TDF, tenofovir disoproxil fumarate.

^a^ Descriptive statistics restricted to sites that had at least 50 participants per individual INSTI drug.

^b^ Median (interquartile range).

^c^ Including persons who identified as MSM and injected drugs.

Among PWH who initiated an INSTI‐based regimen, 28% received RAL, 22% received DTG and 50% received EVG (Table 1B). Persons initiating RAL were older and had lower baseline CD4 compared to those starting EVG or DTG. Additionally, median year of ART initiation was earlier for RAL (2011) compared to DTG (2015) and EVG (2014). The majority of regimens containing RAL and EVG were co‐formulated with TDF, while most DTG‐based regimens were co‐formulated with abacavir.

**Figure 1:**
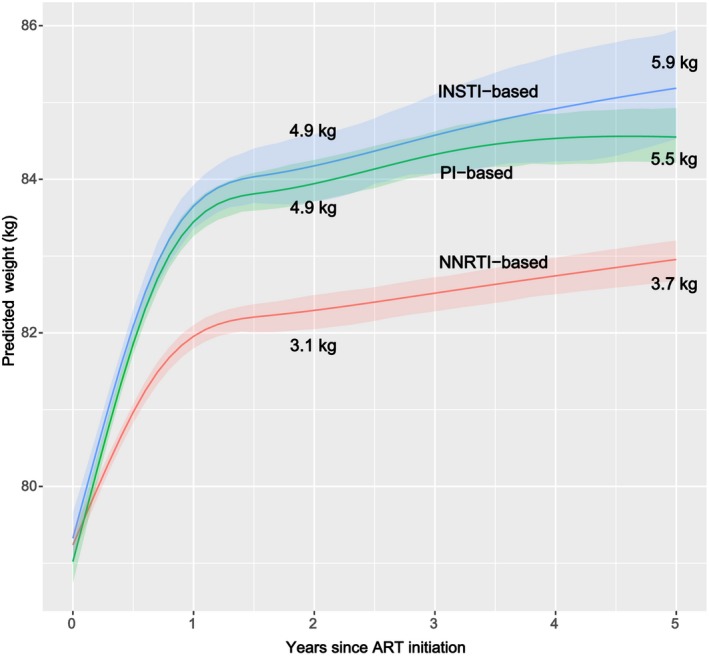
Weight over the first five‐years of ART by regimen class. ART, antiretroviral therapy; INSTI, integrase strand transfer inhibitors; NNRTI, non‐nucleoside reverse transcriptase inhibitors; PI, protease inhibitors.

**Figure 2:**
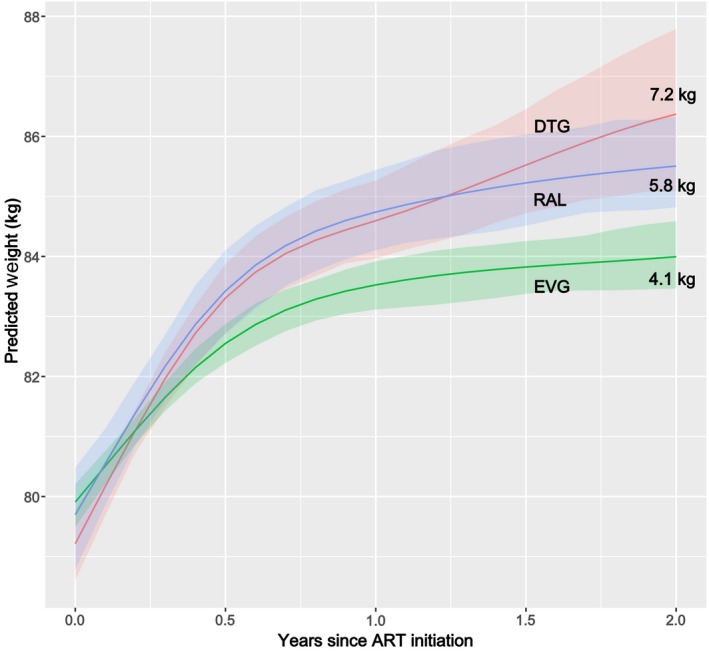
Weight over the first two years of ART among PWH starting INSTI‐based regimens. ART, antiretroviral therapy; DTG, dolutegravir; EVG, elvitegravir; INSTI, integrase strand transfer inhibitors; PWH, persons with HIV; RAL, raltegravir.

Individuals receiving PI‐ and INSTI‐based regimens gained more weight on average than those starting NNRTI‐based regimens (Figure 1); while there was clear separation in the CIs for NNRTIs versus INSTIs and PIs, there was considerable overlap in 95% CIs for the estimated weights of the latter two drug classes. At two and five years, persons on INSTI‐based regimens gained an estimated mean of 4.9 and 5.9kg respectively compared to 4.9 and 5.5kg among persons who received PI‐based regimens, and 3.1 and 3.7kg among NNRTI‐based regimen recipients (Table S1). Within the INSTI class, RAL‐ and DTG‐based regimens were associated with higher estimated mean weight gain than EVG‐based regimens (Figure 2), though intervals for the estimated mean weights on RAL and DTG largely overlapped. At two years, persons starting RAL‐based regimens gained an average of 5.8kg compared to 7.2kg among persons who received DTG‐based regimens, and 4.1kg among EVG‐based regimen recipients. Changes in weight over two years among PWH starting individual INSTI drugs compared to those starting PI‐ or NNRTI‐based regimens is shown in Figure S1. While the estimated mean weight gain for of each of the INSTI drugs was greater than NNRTI‐based regimens at two years, the 95% CIs for weight gain on the individual INSTI drugs largely overlapped with the PI‐based regimens.

**Figure 3:**
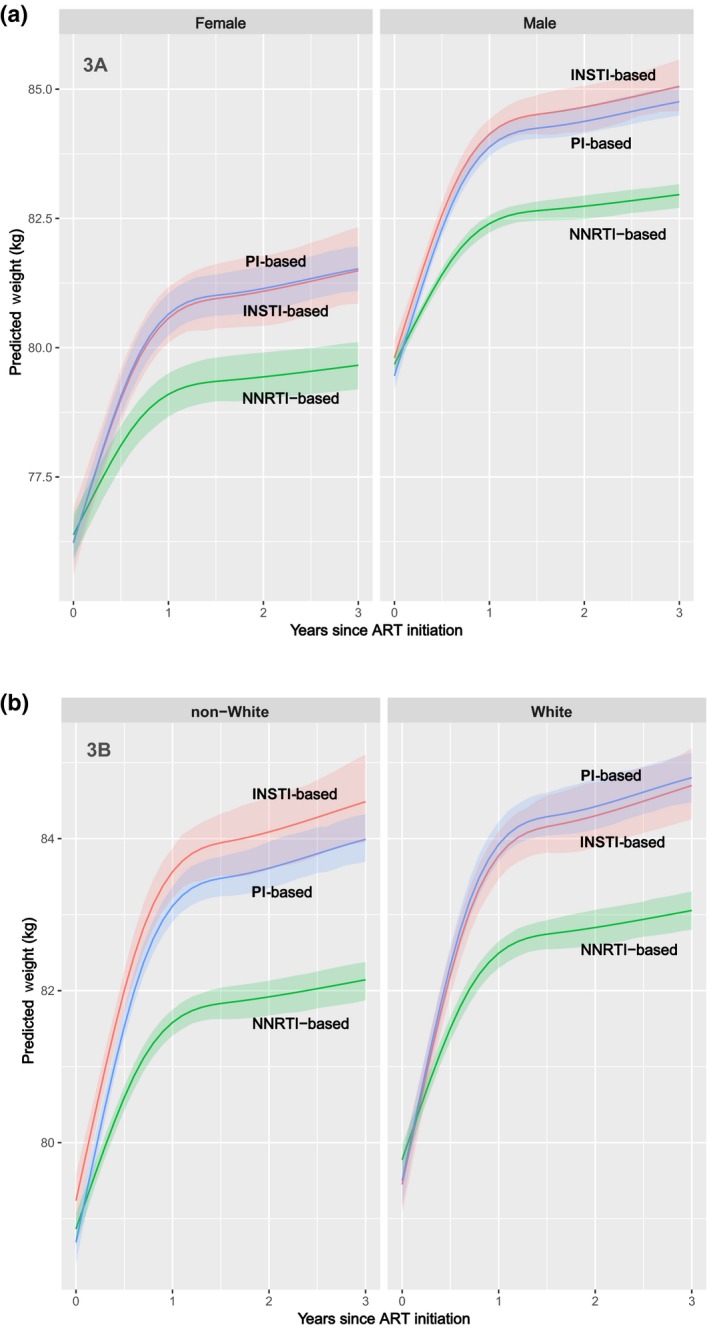
Change in weight over the first three years of ART **(a)** by regimen class and sex **(b)** and by regimen class and dichotomized race. ART, antiretroviral therapy; INSTI, integrase strand transfer inhibitors; NNRTI, non‐nucleoside reverse transcriptase inhibitors; PI, protease inhibitors.

Among male participants weight gain was greater among INSTI‐based ART compared to PI‐based ART, albeit with overlapping 95% CIs, whereas for female participants weight gain was similar for recipients of INSTI‐ and PI‐based regimens (Figure 3a). When the interaction of race and ART regimen type was assessed, INSTI‐associated weight gain was greater than PI‐associated weight gain only for non‐whites whereas INSTI‐associated change was slightly lower than PI‐associated change for whites, though both differences were well within the overlapping Cls (Figure 3b).

**Figure 4:**
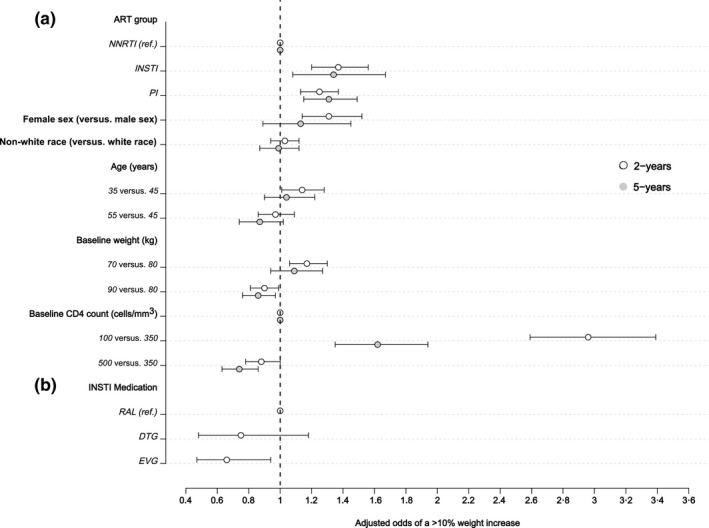
Adjusted odds of a >10% weight increase at two and five years among all participants starting ART **(a)**. Adjusted odds of a >10% weight increase at two years among participants starting INSTI‐based regimens **(b)**. (1) Logistic regression model included in addition to the variables listed: baseline HIV‐1 RNA, cohort site, HIV risk acquisition group and year of ART initiation. (2) Results in (a) are from logistic regression model including all patients with available weight data at year 2 (N = 15,467) and year 5 (N = 6578). (3) Results in (b) are from logistic regression model including only patients on INSTI with available weight data at year 2 (N = 2314). ART, antiretroviral therapy; DTG, dolutegravir; EVG, elvitegravir; INSTI, integrase strand transfer inhibitors; NNRTI, non‐nucleoside reverse transcriptase inhibitors; PI, protease inhibitors; RAL, raltegravir.

Figure 4 reports the adjusted odds of a >10% weight gain, compared to baseline, at two and five years among all persons starting ART (Figure 4a), and at two years for those initiating specific INSTI drug‐based regimens (Figure 4b). Among all ART initiators, the odds of a >10% weight gain were substantially higher for PWH starting PI‐ and INSTI‐based regimens compared to NNRTI‐based regimens at both years 2 and 5. Women and younger persons had higher odds for a >10% weight gain at two years, while at five years the odds continued to be higher for these groups though the associations were attenuated. However, the probability of a >10% weight gain at two years was non‐linear and highest for those with an age of approximately 33 years, which then declined before plateauing between ages 45 and 50 years (Figure S2). Notably, the odds of a >10% weight gain were similar between white and non‐white persons, but a lower baseline weight and CD4 were independent predictors of this degree of weight gain at two and five years. When restricting the analysis to participants starting INSTI‐based regimens, the odds of a >10% weight gain at year 2 were significantly lower for participants receiving EVG compared to RAL.

A substantial proportion of participants transitioned between BMI categories after ART initiation. After three years of ART, 32% of normal‐BMI PWH who initiated an INSTI‐based regimen had become overweight, compared to 29% of those on a PI‐based regimen and 25% on a NNRTI‐based regimen (Figure S3). Rates of transition from overweight to obese after three years of ART use were 28%, 26% and 22% for recipients of INSTI, PI and NNRTI‐based ART respectively. Transitioning to a lower BMI category was observed with all ART regimens, though at a much lower frequency than transitioning to higher BMI categories.

## 4 DISCUSSION

This is the largest observational study to date evaluating associations between initial ART regimens and subsequent weight gain among ART‐naïve PWH. Our findings highlight greater weight gain at two and five years among persons who received INSTI‐based regimens compared to NNRTI‐based regimens. Moreover our findings indicate differential risk for weight gain associated with use of specific INSTI drugs, which was higher among DTG and RAL recipients compared to persons receiving EVG.

Weight gain after ART initiation was often favourably regarded as a “return to health” phenomenon earlier in the HIV epidemic. More recently, with longer survival having become routine among ART‐treated PWH, an increasing proportion of individuals ultimately become overweight or obese after ART initiation. [1, 6, 9, 22, 23]. Among ART‐naïve persons, weight gain after ART initiation tends to be greater among those with lower baseline BMI, higher HIV‐1 viral load and lower CD4 [1]. The origin of this weight gain is multifactorial and may result from a reduction in inflammation‐related catabolism, lower basal energy expenditure, changes in dietary habits or other behaviours (e.g. access to smoking cessation treatment) among other factors [24, 25]. Nevertheless, findings from our study and from similar smaller studies indicate that initial ART agents exert independent effects on weight gain over time as well.

Several demographic characteristics have been associated with increased risk of weight gain among PWH receiving INSTI‐based initial ART regimens. In the prospective ACTG study A5257, the use of RAL with TDF/FTC as an initial regimen was associated with a greater increase in waist circumference at 96 weeks, compared to ritonavir‐boosted darunavir and ritonavir‐boosted atazanavir, each combined with TDF/FTC [18]. Women and persons of black race who received RAL‐based ART experienced greater increases in waist circumference compared to the PI‐based ART recipients. In another study evaluating weight gain following switch to INSTI‐based regimens among virally suppressed ACTG participants in protocols A5001 and A5322, female sex, black race and older age were all associated with greater annualized weight gain after switch to INSTI [26]. Similarly, in the current study we found that female sex, lower baseline weight and lower baseline CD4 were factors associated with significantly higher odds of a >10% weight gain at two and five years after ART initiation.

Among patients receiving INSTI‐based regimens, these findings corroborate our prior report from a smaller Southeastern US cohort in which we described greater weight gain associated with DTG‐based ART regimen use [14], as well as reports from other observational cohorts [15, 16, 17]. Moreover similar disparities in weight gain by initial ART regimen type were noted in a recent 48‐week, open‐label, randomized trial conducted in South Africa comparing EFV/TDF/FTC versus DTG with TDF/FTC or with TAF/FTC, which reported greater increases over 48 weeks in weight and truncal fat among DTG compared to EFV recipients, particularly among persons also receiving TAF. Notably, women were distinctly more prone to truncal fat increases and obesity over 48 weeks compared to men [20]. Lastly, a recent randomized, double‐blind, multi‐centre trial comparing BIC‐ versus DTG‐based ART reported comparable weight gain between the two groups at 96 weeks, but no participants received non‐INSTI‐based ART regimens to serve as comparators [27].

In this study, the overwhelming majority of participants received ART regimens containing TDF or abacavir, and prior studies have not observed substantially different weight changes with use of these two NRTIs [28, 29]. Notably, <1% of participants received an ART regimen containing TAF. The use of TAF, approved in the US in November 2015, is rapidly increasing given the lower incidence of adverse bone and renal effects compared to TDF. Recent data from pooled clinical trials and other studies indicate that TAF use predisposes to weight gain independent of concomitant INSTI use [20, 30, 31]. The very low number of participants receiving TAF is both a strength and a limitation of our study. By essentially excluding TAF recipients our analysis was able to identify more fully the effects of INSTI drug use on weight change after ART initiation, though additional analyses will be needed in the future to assess the potential contribution of TAF to ART‐associated weight gain.

Some caution is warranted in interpreting the findings from this study, as our observational cohort was drawn from multiple sites in North America and results may not be generalizable to all PWH, particularly in non‐US/Canadian settings. Additionally, based on the Food and Drug Administration approval date for DTG, we limited the analysis of variation in weight gain by individual INSTI drugs to two years, and our analysis period preceded the introduction of BIC. Moreover our analysis did not adjust for exposure to non‐HIV medications associated with weight gain (e.g. hormonal and psychotropic drugs) nor did we adjust for pregnancy, as these data were not routinely collected in the cohort. Furthermore, only 13% of participants were females; therefore, we might be misrepresenting the population‐level impact of weight gain in the target population of HIV positive individuals initiating ART in the United States and Canada during this period – especially as several studies have shown ART‐associated weight gain to be a greater problem among females [20, 30]. Finally, we did not assess clinical outcomes, such as metabolic or cardiovascular disease incidence or progression, and future studies will be needed to determine the impact of the observed weight changes on the health of PWH. Further studies are also needed to evaluate the mechanism of weight gain among patients starting INSTI‐based regimens. With the current available evidence, it remains unclear whether the weight gain is secondary to off‐target effects of these drugs and a toxicity, or whether it’s due to the higher effectiveness, safety and tolerability of these medications.

## 5 CONCLUSION

In this multisite cohort of over 20,000 diverse treatment‐naïve PWH across North America who initiated ART, we found that the use of INSTI‐based ART regimens was associated with higher weight gain up to five years after treatment initiation compared to NNRTI‐based regimens, primarily driven by the use of DTG‐ and RAL‐containing regimens. Additionally, INSTI‐based ART was associated with comparable weight gain to PI‐based regimens. ART‐treated PWH have a high prevalence of obesity and are already at greater risk for metabolic and cardiovascular comorbidities; closely monitoring changes in weight after the initiation of INSTI‐class drugs may identify patients who could benefit from weight loss interventions and closer scrutiny of associated cardio‐metabolic risks.

## COMPETING INTERESTS

KA serves on an advisory board for TrioHealth. KB receives research funding from Gilead Sciences, and has served as a scientific advisor for Gilead Sciences and Theratechnologies. HC has served on an advisory board for ViiV. JG has served on the National HIV Advisory Boards of Merck, Gilead and Viivhealth. JK receives research funding from Gilead Sciences, and has served as a scientific advisor for Gilead Sciences and Theratechnologies. JLake receives research funding from Gilead Sciences and is a consultant to Gilead Sciences and Merck. MS receives research funding from Gilead Sciences. FP is a consultant and/or on the Speakers’ Bureau for Gilead Sciences, Janssen, ViiV and Merck. JT is a consultant for Gilead Sciences, has served as a scientific advisor to AbbVie, Clearside and Santen, and has grant support from Allergan and NEI. SG, MH, CJ, GK, JLi, VL, JM, RM, WCM, CR, PR, TS, AW and CW report no conflicts of interest.

## AUTHORS’ CONTRIBUTIONS

KB, CJ, PR, JLake and JK developed the study design and analysis plan, which was reviewed, modified and approved by RM, KA, FP, JG, CR, SG, MH, JM, JLi, CW, AW, VL, HC, JT, MS, GK and WCM. CJ and PR conducted the statistical analysis with periodic input from JK, JLake, KB and TS. KB, JK, JLake, TS, CJ and PR drafted the initial version of the manuscript, which was then revised by RM, KA, FP, JG, CR, SG, MH, JM, JLi, CW, AW, VL, HC, JT, MS, GK and WCM over several iterations. All authors approved the final manuscript.

## Supporting information


**Figure S1. **Changes in weight within the first two‐years of ART initiation among PWH starting different INSTI‐ drugs compared to those starting PI‐ or NNRTI‐based regimens.

**Figure S2. **Predicted probability of >10% weight gain after two years of ART by age and regimen class.

**Figure S3. **Transition between the BMI categories of underweight (<18.5kg/m^2^), normal BMI (18.5 to 24.9kg/m^2^), overweight (25.0 to 29.9kg/m^2^) and obese (≥30kg/m^2^) over three years of ART by regimen class.

**Table S1. **Predicted weight (95% confidence interval) and predicted change in weight compared to baseline for persons with HIV starting different ART regimens.

## References

[1] Koethe JR , Jenkins CA , Lau B , Shepherd BE , Justice AC , Tate JP , et al. Rising obesity prevalence and weight gain among adults starting antiretroviral therapy in the United States and Canada. AIDS Res Hum Retroviruses. 2016;32(1):50–8. 26352511 10.1089/aid.2015.0147 PMC4692122

[2] Lakey WC , Yang LY , Yancy W , Chow SC , Hicks CB . From wasting to obesity: initial antiretroviral therapy and weight gain in HIV‐infected persons. AIDS Res Hum Retroviruses. 2013;29(3):435–40. 23072344 10.1089/aid.2012.0234 PMC3581041

[3] Yuh B , Tate J , Butt AA , Crothers K , Freiberg M , Leaf D , et al. Weight change after antiretroviral therapy and mortality. Clin Infect Dis. 2015;60(12):1852–9. 25761868 10.1093/cid/civ192 PMC4542664

[4] Madec Y , Szumilin E , Genevier C , Ferradini L , Balkan S , Pujades M , et al. Weight gain at 3 months of antiretroviral therapy is strongly associated with survival: evidence from two developing countries. AIDS. 2009;27(7):853–61. 10.1097/QAD.0b013e32832913ee 19287299

[5] Koethe JR , Lukusa A , Giganti MJ , Chi BH , Nyirenda CK , Limbada MI , et al. Association between weight gain and clinical outcomes among malnourished adults initiating antiretroviral therapy in Lusaka, Zambia. J Acquir Immune Defic Syndr. 2010;53(4):507–13. 19730111 10.1097/QAI.0b013e3181b32baf PMC3749827

[6] Herrin M , Tate JP , Akgun KM , Butt AA , Crothers K , Freiberg MS , et al. Weight gain and incident diabetes among HIV‐infected veterans initiating antiretroviral therapy compared with uninfected individuals. J Acquir Immune Defic Syndr. 2016;73(2):228–36. 27171741 10.1097/QAI.0000000000001071 PMC5023454

[7] Sattler FR , He J , Letendre S , Wilson C , Sanders C , Heaton R , et al. Abdominal obesity contributes to neurocognitive impairment in HIV‐infected patients with increased inflammation and immune activation. J Acquir Immune Defic Syndr. 2015;68(3):281–8. 25469522 10.1097/QAI.0000000000000458 PMC4551458

[8] Kim DJ , Westfall AO , Chamot E , Willig AL , Mugavero MJ , Ritchie C , et al. Multimorbidity patterns in HIV‐infected patients: the role of obesity in chronic disease clustering. J Acquir Immune Defic Syndr. 2012;61(5):600–5. 23023101 10.1097/QAI.0b013e31827303d5 PMC3508375

[9] Butt AA , McGinnis K , Rodriguez‐Barradas MC , Crystal S , Simberkoff M , Goetz MB , et al. HIV infection and the risk of diabetes mellitus. AIDS. 2009;23(10):1227–34. 19444074 10.1097/QAD.0b013e32832bd7af PMC2752953

[10] Podany AT , Scarsi KK , Fletcher CV . Comparative clinical pharmacokinetics and pharmacodynamics of HIV‐1 integrase strand transfer inhibitors. Clin Pharmacokinet. 2017;56(1):25–40. 27317415 10.1007/s40262-016-0424-1 PMC5164870

[11] Park TE , Mohamed A , Kalabalik J , Sharma R . Review of integrase strand transfer inhibitors for the treatment of human immunodeficiency virus infection. Expert Rev Anti Infect Ther. 2015;13(10):1195–212. 26293294 10.1586/14787210.2015.1075393

[12] Panel on Antiretroviral Guidelines for Adults and Adolescents . Guidelines for the use of antiretroviral agents in HIV‐1‐infected adults and adolescents. Department of Health and Human Services [cited 2020 Feb 22]. Available from: http://www.aidsinfo.nih.gov/ContentFiles/AdultandAdolescentGL.pdf

[13] Bakal DR , Coelho LE , Luz PM , Clark JL , De Boni RB , Cardoso SW , et al. Obesity following ART initiation is common and influenced by both traditional and HIV‐/ART‐specific risk factors. J Antimicrob Chemother. 2018;73(8):2177–85. 29722811 10.1093/jac/dky145 PMC6054231

[14] Bourgi K , Rebeiro PF , Turner M , Castilho JL , Hulgan T , Raffanti SP , et al. Greater weight gain in treatment naïve persons starting dolutegravir‐based antiretroviral therapy. Clin Infect Dis. 2019;70:1267–1274. 10.1093/cid/ciz407 PMC8205610 31100116

[15] Menard A , Meddeb L , Tissot‐Dupont H , Ravaux I , Dhiver C , Mokhtari S , et al. Dolutegravir and weight gain: an unexpected bothering side effect? AIDS. 2017;31(10):1499–500. 28574967 10.1097/QAD.0000000000001495

[16] Rizzardo S , Lanzafame M , Lattuada E , Luise D , Vincenzi M , Tacconelli E , et al. Dolutegravir monotherapy and body weight gain in antiretroviral naive patients. AIDS. 2019;33(10):1673–4. 31305333 10.1097/QAD.0000000000002245

[17] Norwood J , Turner M , Bofill C , Rebeiro P , Shepherd B , Bebawy S , et al. Weight gain in persons with HIV switched from efavirenz‐based to integrase strand transfer inhibitor‐based regimens. J Acquir Immune Defic Syndr. 2017;76(5):527–31. 28825943 10.1097/QAI.0000000000001525 PMC5680113

[18] Bhagwat P , Ofotokun I , McComsey GA , Brown TT , Moser C , Sugar CA , et al. Changes in waist circumference in HIV‐infected individuals initiating a raltegravir or protease inhibitor regimen: effects of sex and race. Open Forum Infect Dis. 2018;5(11):ofy201. 30465010 10.1093/ofid/ofy201 PMC6239079

[19] Bhagwat P , Ofotokun I , McComsey G , Brown T , Moser C , Sugar C , et al.Abstract 695: predictors of severe weight/body mass index gain following antiretroviral initiation. Conference on retroviruses and opportunistic infections, Seattle (WA); 2017 [cited 2020 Feb 22]. Available from: http://www.croiconference.org/sessions/predictors-severe-weightbody-mass-index-gain-following-antiretroviral-initiation-0

[20] Venter WD , Moorhouse M , Sokhela S , Fairlie L , Mashabane N , Masenya M , et al. Dolutegravir plus two different prodrugs of tenofovir to treat HIV. N Engl J Med. 2019;381(9):803–15. 31339677 10.1056/NEJMoa1902824

[21] Gange SJ , Kitahata MM , Saag MS , Bangsberg DR , Bosch RJ , Brooks JT , et al. Cohort profile: the North American AIDS Cohort Collaboration on Research and Design (NA‐ACCORD). Int J Epidemiol. 2007;36(2):294–301. 17213214 10.1093/ije/dyl286 PMC2820873

[22] Koethe JR , Grome H , Jenkins CA , Kalams SA , Sterling TR . The metabolic and cardiovascular consequences of obesity in persons with HIV on long‐term antiretroviral therapy. AIDS. 2016;30(1):83–91. 26418084 10.1097/QAD.0000000000000893 PMC4703444

[23] Freiberg MS , Chang CC , Kuller LH , Skanderson M , Lowy E , Kraemer KL , et al. HIV infection and the risk of acute myocardial infarction. JAMA Intern Med. 2013;173(8):614–22. 23459863 10.1001/jamainternmed.2013.3728 PMC4766798

[24] Leite LM , Sampaio ADMM . Progression to overweight, obesity and associated factors after antiretroviral therapy initiation among Brazilian persons with HIV/AIDS. Nutr Hosp. 2010;25(4):635–40. 20694301

[25] Mave V , Erlandson KM , Gupte N , Balagopal A , Asmuth DM , Campbell TB , et al. Inflammation and change in body weight with antiretroviral therapy initiation in a multinational cohort of HIV‐infected adults. J Infect Dis. 2016;214(1):65–72. 26962236 10.1093/infdis/jiw096 PMC4907416

[26] Lake JE , Wu K , Bares SH , Debroy P , Godfrey C , Koethe JR , et al. Risk Factors for Weight Gain Following Switch to Integrase Inhibitor‐Based Antiretroviral Therapy. Clin Infect Dis. 2020. pii: ciaa177. doi: 10.1093/cid/ciaa177. [Epub ahead of print] PMC7713693 32099991

[27] Wohl DA , Yazdanpanah Y , Baumgarten A , Clarke A , Thompson MA , Brinson C , et al. Bictegravir combined with emtricitabine and tenofovir alafenamide versus dolutegravir, abacavir, and lamivudine for initial treatment of HIV‐1 infection: week 96 results from a randomised, double‐blind, multicentre, phase 3, non‐inferiority trial. Lancet HIV. 2019;6(6):e355–e63. 31068270 10.1016/S2352-3018(19)30077-3

[28] Erlandson KM , Kitch D , Tierney C , Sax PE , Daar ES , Tebas P , et al. Weight and lean body mass change with antiretroviral initiation and impact on bone mineral density: AIDS clinical trials group study A5224s. AIDS. 2013;27(13):2069–79. 24384588 10.1097/QAD.0b013e328361d25d PMC3966569

[29] Erlandson KM , Kitch D , Tierney C , Sax PE , Daar ES , Melbourne KM , et al. Impact of randomized antiretroviral therapy initiation on glucose metabolism: AIDS clinical trials group study A5224s. AIDS. 2014;28(10):1451–61. 24637543 10.1097/QAD.0000000000000266 PMC4167596

[30] Sax PE , Erlandson KM , Lake JE , Mccomsey GA , Orkin C , Esser S , et al. Weight gain following initiation of antiretroviral therapy: risk factors in randomized comparative clinical trials. Clin Infect Dis. 2019;pii:ciz999. 10.1093/cid/ciz999 PMC7486849 31606734

[31] Gomez M , Seybold U , Roider J , Harter G , Bogner JR . A retrospective analysis of weight changes in HIV‐positive patients switching from a tenofovir disoproxil fumarate (TDF)‐ to a tenofovir alafenamide fumarate (TAF)‐containing treatment regimen in one German university hospital in 2015–2017. Infection. 2019;47(1):95–102. 30269210 10.1007/s15010-018-1227-0 PMC7384998

