# Corrigenda

**DOI:** 10.1002/jia2.25549

**Published:** 2020-07-27

Regarding the paper “Weight gain among treatment‐naïve persons with HIV starting integrase inhibitors compared to non‐nucleoside reverse transcriptase inhibitors or protease inhibitors in a large observational cohort in the United States and Canada” by Kassem Bourgi, Cathy A Jenkins, Peter F Rebeiro, Frank Palella, Richard D Moore, Keri N Altoff, John Gill, Charles S Rabkin, Stephen J Gange, Michael A Horberg, Joseph Margolick, Jun Li, Cherise Wong, Amanda Willig, Viviane D Lima, Heidi Crane, Jennifer Thorne, Michael Silverberg, Gregory Kirk, William C Mathews, Timothy R Sterling, Jordan Lake and, John R Koethe for the North American AIDS Cohort Collaboration on Research and Design (NA‐ACCORD)

Published in the *Journal of the International AIDS Society*, Citation: *Journal of the International AIDS Society* 2020, 23: 25484. https://doi.org/10.1002/jia2.25484

The authors noticed the following errors in the author byline and should have been corrected.

1. The author name, “Keri N Altoff” was misspelt and should have been corrected to “Keri N Althoff”.
2. Bryan Shepherd was omitted and should have been added to the list of authors.

The author byline should have read:

Kassem Bourgi^1,2^, Cathy A. Jenkins^1^, Peter F. Rebeiro^1^, Bryan E. Shepherd^1^, Frank Palella^3^, Richard D. Moore^4^, Keri N. Althoff^4^, John Gill^5^, Charles S. Rabkin^6^, Stephen J. Gange^4^, Michael A. Horberg^7^, Joseph Margolick^4^, Jun Li^8^, Cherise Wong^4^, Amanda Willig^9^, Viviane D. Lima^10^, Heidi Crane^11^, Jennifer Thorne^4^, Michael J. Silverberg^12^, Gregory Kirk^4^, William Christopher Mathews^13^, Timothy R. Sterling^1^, Jordan Lake^14^ and John R. Koethe^1,15,§^ for the North American AIDS Cohort Collaboration on Research and Design (NA‐ACCORD)

^1^Vanderbilt University Medical Center, Nashville, Tennessee, USA; ^2^Indiana University School of Medicine, Indianapolis, Indiana, USA; ^3^Northwestern University Feinberg School of Medicine, Chicago, IL, USA; ^4^Johns Hopkins University, Baltimore, Maryland, USA; ^5^University of Calgary, Calgary, Alberta, Canada; ^6^National Cancer Institute, Bethesda, Maryland, USA; ^7^Mid‐Atlantic Permanente Research Institute, Kaiser Permanente Mid‐Atlantic States, Rockville, Maryland, USA; ^8^Centers for Disease Control and Prevention, Atlanta, Georgia, USA; ^9^University of Alabama at Birmingham, Birmingham, Alabama, USA; ^10^University of British Columbia, Vancouver, British Columbia, Canada; ^11^University of Washington, Seattle, Washington, USA; ^12^Kaiser Permanente Division of Research, Kaiser Permanente Northern California, Oakland, California, USA; ^13^University of California San Diego, San Diego, California, USA; ^14^University of Texas Health Science Center at Houston, Houston, Texas, USA; ^15^Veterans Affairs Tennessee Valley Healthcare System, Nashville, Tennessee, USA

This article has been corrected online.

