# Supplementary material for: Weight gain among treatment‐naïve persons with HIV starting integrase inhibitors compared to non‐nucleoside reverse transcriptase inhibitors or protease inhibitors in a large observational cohort in the United States and Canada
Source: J Int AIDS Soc. 2020 Apr 15;23(4):e25484. doi: 10.1002/jia2.25484 (PMC7159248; doi:10.1002/jia2.25484)

Supplemental Figure 1: Changes in weight within the first 2-years of ART initiation among persons with HIV starting different INSTI- drugs compared to those starting PI- or NNRTI-based regimens


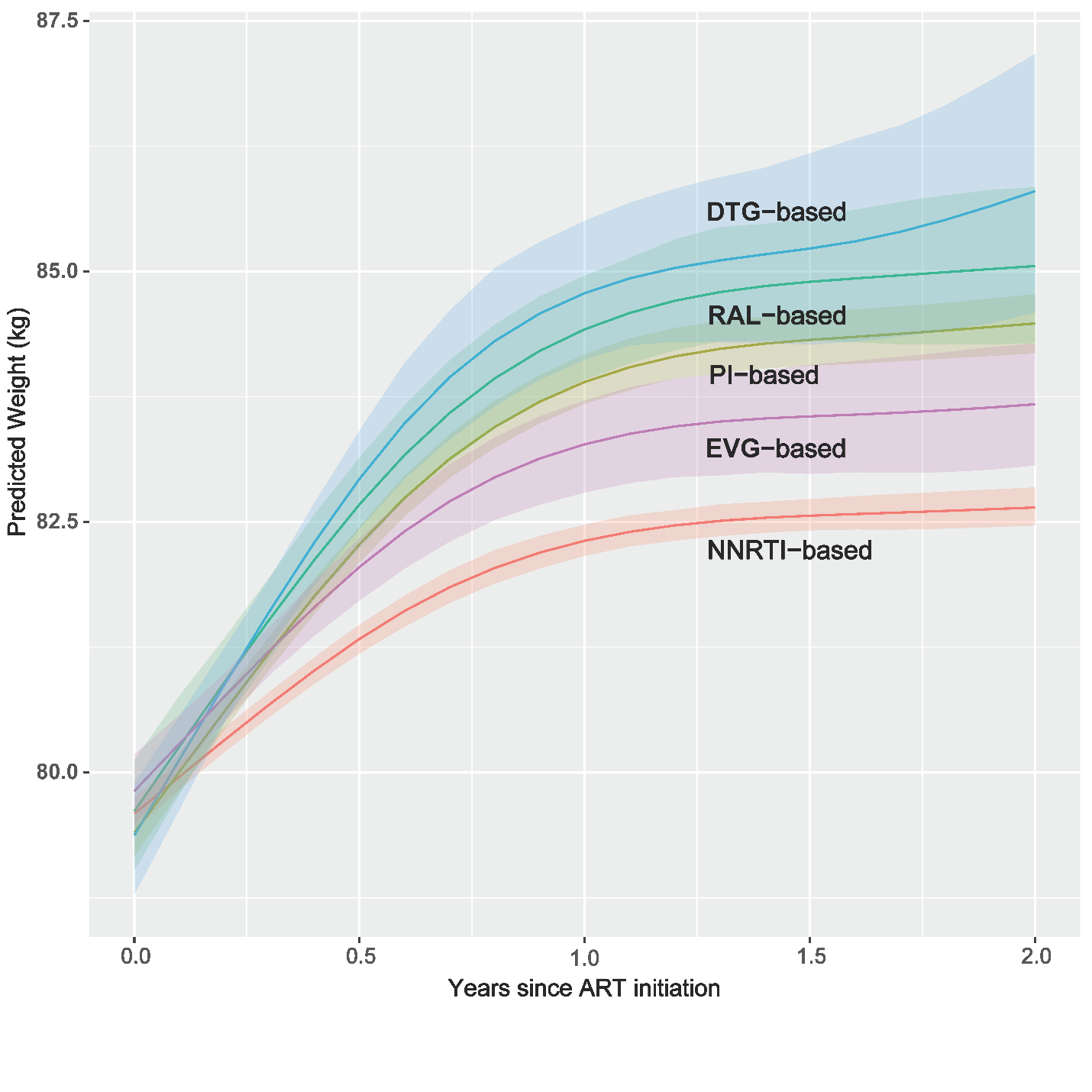

Supplement: Supplementary file 1 — Figure S1. Changes in weight within the first two‐years of ART initiation among PWH starting different INSTI‐ drugs compared to those starting PI‐ or NNRTI‐based regimens. [file JIA2-23-e25484-s001.docx]
