# Supplementary material for: Weight gain among treatment‐naïve persons with HIV starting integrase inhibitors compared to non‐nucleoside reverse transcriptase inhibitors or protease inhibitors in a large observational cohort in the United States and Canada
Source: J Int AIDS Soc. 2020 Apr 15;23(4):e25484. doi: 10.1002/jia2.25484 (PMC7159248; doi:10.1002/jia2.25484)

Supplemental Figure 2: Predicted probability of >10% weight gain after 2 years of ART by age and regimen class


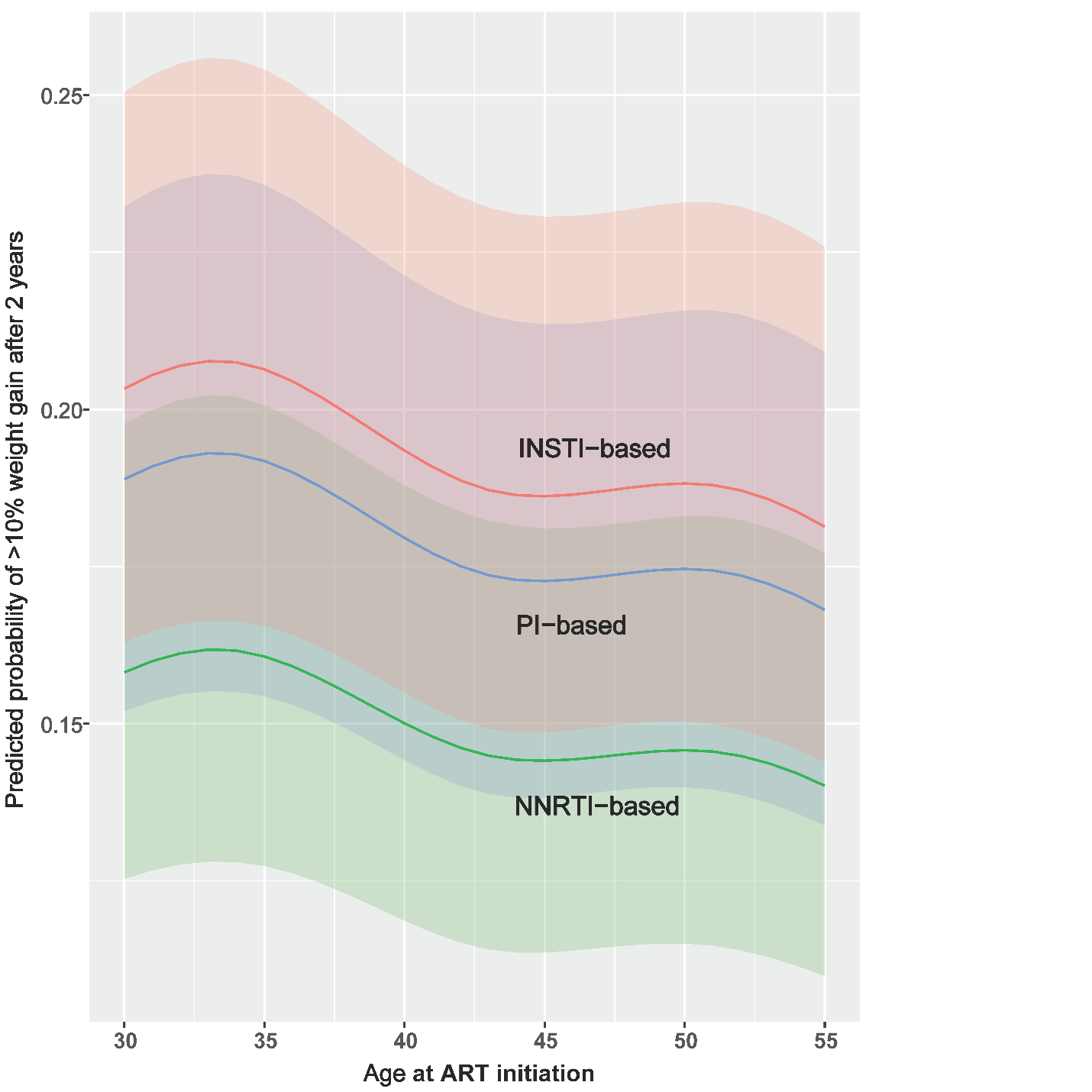

Supplement: Supplementary file 2 — Figure S2. Predicted probability of >10% weight gain after two years of ART by age and regimen class. [file JIA2-23-e25484-s002.docx]
