# Supplementary material for: Weight gain among treatment‐naïve persons with HIV starting integrase inhibitors compared to non‐nucleoside reverse transcriptase inhibitors or protease inhibitors in a large observational cohort in the United States and Canada
Source: J Int AIDS Soc. 2020 Apr 15;23(4):e25484. doi: 10.1002/jia2.25484 (PMC7159248; doi:10.1002/jia2.25484)

Supplemental Figure 3

Transition between the body mass index categories of underweight (<18.5 kg/m^2^), normal BMI (18.5 – 24.9 kg/m^2^), overweight (25.0 – 29.9 kg/m^2^), and obese (>=30kg/m^2^) over 3 years of ART by regimen class

3A: INSTI-based regimens


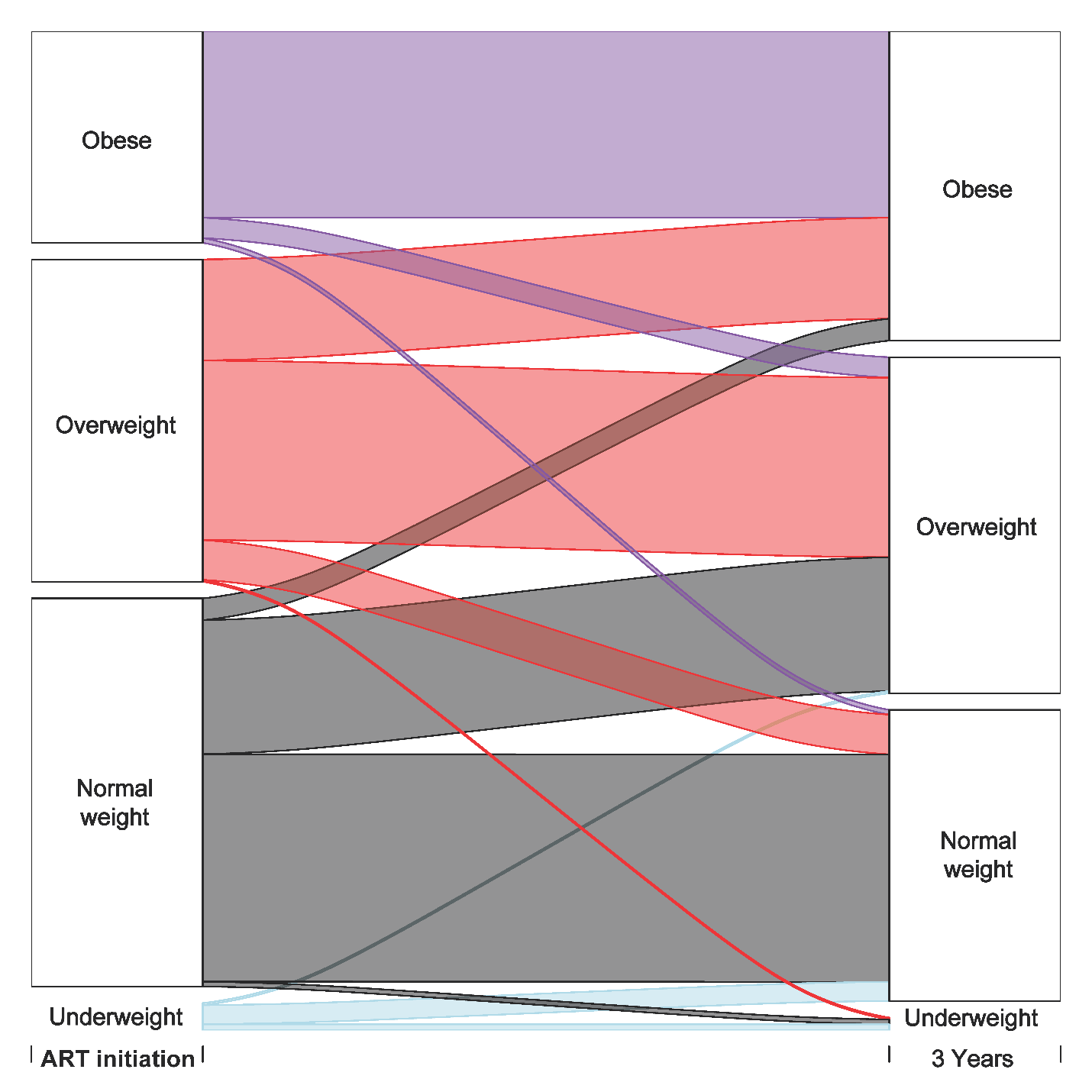


3B: PI-based regimens


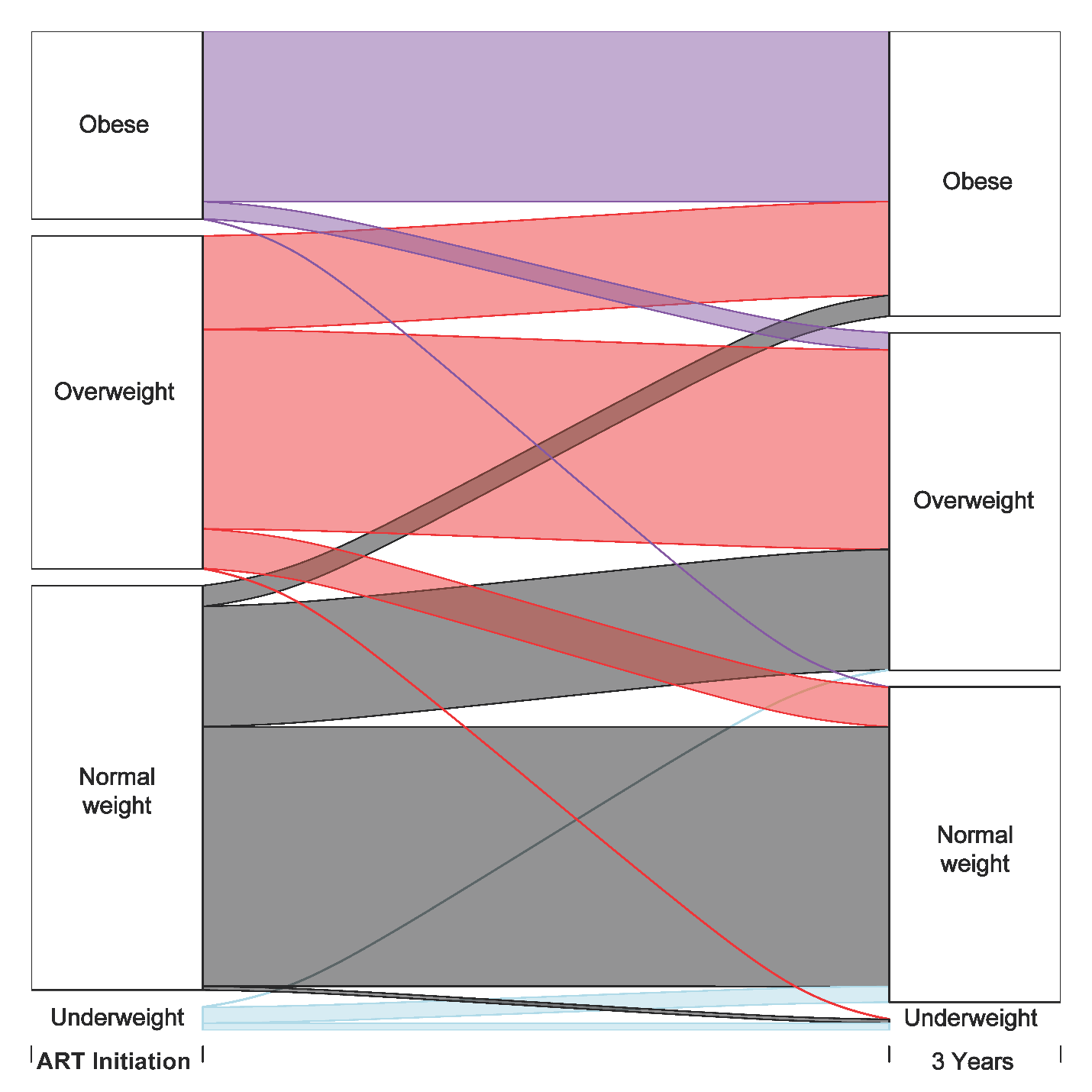


3C: NNRTI-based regimens


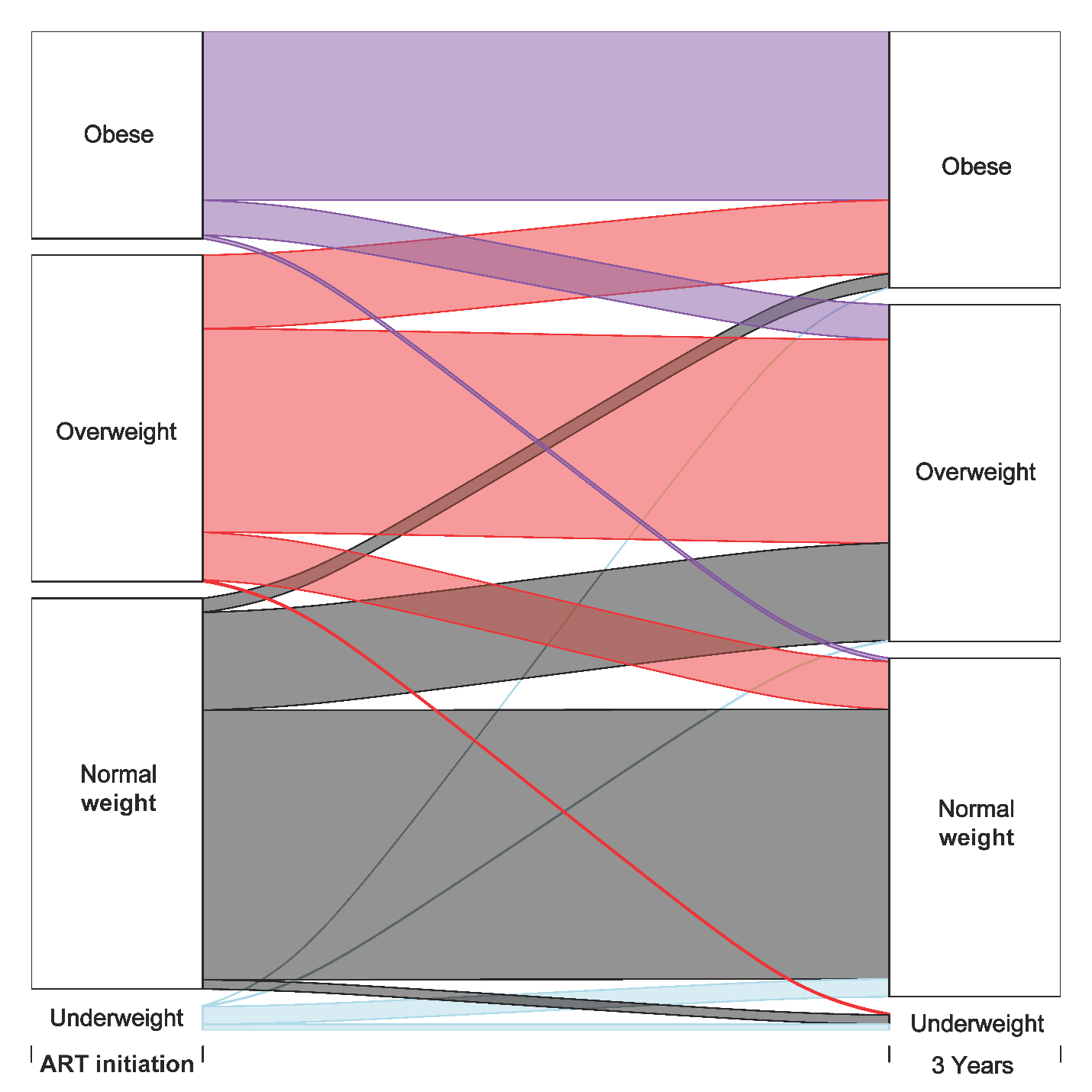

Supplement: Supplementary file 3 — Figure S3. Transition between the BMI categories of underweight (<18.5kg/m2), normal BMI (18.5 to 24.9kg/m2), overweight (25.0 to 29.9kg/m2) and obese (≥30kg/m2) over three years of ART by regimen class. [file JIA2-23-e25484-s003.docx]
