# Supplementary material for: Weight gain among treatment‐naïve persons with HIV starting integrase inhibitors compared to non‐nucleoside reverse transcriptase inhibitors or protease inhibitors in a large observational cohort in the United States and Canada
Source: J Int AIDS Soc. 2020 Apr 15;23(4):e25484. doi: 10.1002/jia2.25484 (PMC7159248; doi:10.1002/jia2.25484)
Supplement: Supplementary file 4 — Table S1. Predicted weight (95% confidence interval) and predicted change in weight compared to baseline for persons with HIV starting different ART regimens. [file JIA2-23-e25484-s004.docx]

## Supplemental Table

Predicted weight (95% confidence interval) and predicted change in weight compared to baseline at different time points for PWH starting different ART regimens.

| **Time since ART initiation (years)** | **NNRTI-based regimen** | **PI- based regimen** | **INSTI-based regimen** | | | |
| --- | --- | --- | --- | --- | --- | --- |
|  |  |  | **All INSTI** | **RAL** | **DTG** | **EVG** |
| **0** | **79.2**  *(79.1, 79.4)*  0 | **79.0**  *(78.7, 79.3)*  0 | **79.3**  *(79.0, 79.7)*  0 | **79.7**  *(78.8, 80.5)*  0 | **79.2**  *(78.6, 79.8)*  0 | **79.9**  *(79.5, 80.2)*  0 |
| **0.5** | **81.0**  *(80.8, 81.1)*  +1.8 | **81.9**  *(81.7, 82.0)*  +2.9 | **82.1**  *(81.9, 82.3)*  +2.8 | **83.4**  *(82.7, 84.1)*  +3.7 | **83.3**  *(82.7, 83.9)*  +4.1 | **82.6**  *(82.2, 82.9)*  +2.7 |
| **1** | **82.0**  *(81.8, 82.1)*  +2.8 | **83.4**  *(83.3, 83.7)*  +4.4 | **83.7**  *(83.4, 83.9)*  +4.4 | **84.7**  *(84.1, 85.4)*  +5.0 | **84.6**  *(84.0, 85.3)*  +5.4 | **83.5**  *(83.1, 83.9)*  +3.6 |
| **2** | **82.3**  *(82.0, 82.5)*  +3.1 | **83.9**  *(83.7, 84.3)*  +4.9 | **84.2**  *(83.7, 84.6)*  +4.9 | **85.5**  *(84.8, 86.3)*  +5.8 | **86.4**  *(85.1, 87.8)*  +7.2 | **84.0**  *(83.5, 84.6)*  +4.1 |
| **3** | **82.5**  *(82.3, 82.7)*  +3.3 | **84.3**  *(84.1, 84.6)*  +5.3 | **84.6**  *(84.1, 85.1)*  +5.3 |  | | |
| **4** | **82.7**  *(82.5, 83.0)*  +3.5 | **84.5**  *(84.2, 84.9)*  +5.5 | **84.9**  *(84.2, 85.6)*  +5.6 |  |  |  |
| **5** | **83.0**  *(82.7, 83.2)*  +3.8 | **84.6**  *(84.2, 84.9)*  +5.6 | **85.2**  *(84.5, 85.9)*  +5.9 |  |  |  |
